# Design of a High-Sensitivity Dimeric G-Quadruplex/Hemin DNAzyme Biosensor for Norovirus Detection

**DOI:** 10.3390/molecules26237352

**Published:** 2021-12-03

**Authors:** Yun Zhang, Xinao Ma, Jingtian Zhang, Feixian Luo, Wenshu Wang, Xiaojie Cui

**Affiliations:** 1 College of Life and Environmental Sciences, Minzu University of China, Beijing 100081, China; 19010963@muc.edu.cn (Y.Z.); 19140125@muc.edu.cn (X.M.); 20160023@muc.edu.cn (J.Z.); luofeixian@muc.edu.cn (F.L.); wangws@muc.edu.cn (W.W.); 2 Key Laboratory of Ecology and Environment in Minority Areas (Minzu University of China), National Ethnic Affairs Commission, Beijing 100081, China

**Keywords:** G-quadruplex, dimeric, DNAzyme, biosensor, norovirus

## Abstract

G-quadruplexes can bind with hemin to form peroxidase-like DNAzymes that are widely used in the design of biosensors. However, the catalytic activity of G-quadruplex/hemin DNAzyme is relatively low compared with natural peroxidase, which hampers its sensitivity and, thus, its application in the detection of nucleic acids. In this study, we developed a high-sensitivity biosensor targeting norovirus nucleic acids through rationally introducing a dimeric G-quadruplex structure into the DNAzyme. In this strategy, two separate molecular beacons each having a G-quadruplex-forming sequence embedded in the stem structure are brought together through hybridization with a target DNA strand, and thus forms a three-way junction architecture and allows a dimeric G-quadruplex to form, which, upon binding with hemin, has a synergistic enhancement of catalytic activities. This provides a high-sensitivity colorimetric readout by the catalyzing H_2_O_2_-mediated oxidation of 2,2′-azino-bis(3-ethylbenzothiazoline -6-sulfonic acid) diammonium salt (ABTS). Up to 10 nM of target DNA can be detected through colorimetric observation with the naked eye using our strategy. Hence, our approach provides a non-amplifying, non-labeling, simple-operating, cost-effective colorimetric biosensing method for target nucleic acids, such as norovirus-conserved sequence detection, and highlights the further implication of higher-order multimerized G-quadruplex structures in the design of high-sensitivity biosensors.

## 1. Introduction

G-quadruplex is a higher-order DNA or RNA structure formed by guanine-rich nucleic acids [1]. Besides its variable biological relevance [2,3], G-quadruplexes are found to be able to bind with hemin to form a kind of peroxidase-like enzyme called G-quadruplex/hemin DNAzyme, which can catalyze the oxidation of substrates such as ABTS, TMB (tetramethylbenzidine) and luminol by H_2_O_2_, and hence, generates a visible color change. Because of its stable chemical properties, easy modification and programming accessibility, G-quadruplex/hemin DNAzymes are widely used in the design of biosensors for detecting target nucleic acids [4,5]. However, the sensitivity of the G-quadruplex/hemin DNAzyme biosensor it relatively low, which hampers its further application [5].

The sensitivity of the G-quadruplex/hemin DNAzyme biosensor relies on the catalytic activity of the DNAzyme. Previous studies have shown that the structure of the G-quadruplex has a crucial impact on the catalytic efficiency. For example, the DNAzyme formed by parallel G-quadruplex has a much higher catalytic efficiency than that formed by antiparallel G-quadruplex [6], and that formed by G-quadruplex with adenosine at the 3′-end shows an enhanced catalytic activity [7], while that formed with adenosines at both the 3′- and 5′-ends can efficiently catalyze the oxidation even at 95 °C [8]. Besides, two or more individual G-quadruplexes stacked on top of each other to form a multimeric G-quadruplex have a synergistic enhancement effect on the catalytic activity of the DNAzyme [9,10,11]. Several groups have found that the DNAzyme bearing multimeric G-quadruplex is more active than the sum of activities of an equal number of DNAzymes bearing monomeric G-quadruplex, probably because the multimeric G-quadruplex provides high-activity sites for both hemin and the oxidant H_2_O_2_ [9,10,11]. Therefore, introducing a multimeric G-quadruplex structure into the DNAzyme-based biosensor could be a promising strategy to improve its sensitivity. However, so far, in the design of biosensors based on G-quadruplex/hemin DNAzyme, multiple protein enzymes are usually applied to amplify the target nucleic acids, and hence, enrich the G-quadruplex units so as to improve their sensitivity [12,13,14,15,16], while the synergistic enhancement of catalytic activities caused by the multimerization of G-quadruplex units is neglected.

Norovirus is a contagious virus that is the most common cause of acute gastroenteritis [17]. Although noroviruses affect people of all ages globally, infection incidence rates are highest among young children under five years old, especially in developing countries [18]. It is reported that the direct health system costs of norovirus are around $4.2 billion per year globally [19]. In China, norovirus infection outbreaks show a constantly increasing trend and have brought a considerable burden to public healthcare [20]. It is known that noroviruses are 7.5–7.7 kb positive-stranded RNA viruses and currently can be classified into seven (GI to GVII) genogroups with GI and GII being the most common cause of human diseases [21]. Right now, molecular tests such as reverse-transcriptase polymerase chain reaction (RT-PCR) and quantitative, real time RT-PCR (RT-qPCR) are the gold standards for norovirus detection, but these methods require expensive facilities and trained expertise to conduct the assay and, thus, may not be applicable in underdeveloped areas [21]. Other detection methods such as the enzyme immunoassay [22], immunochromatographic lateral flow assays [23] and drop microfluidic assays [24] are also limited by relatively high costs. Based on the DNAzyme biosensor strategy, Kim’s group [25] developed a colorimetric detection method for norovirus in which the G-quadruplex reporter is blocked and the signal is switched “off” through PCR amplification of the target. While this method is able to detect up to a single copy of norovirus, it is PCR-dependent and requires protein enzymes, which increase the labor and cost involved.

**Scheme 1:**
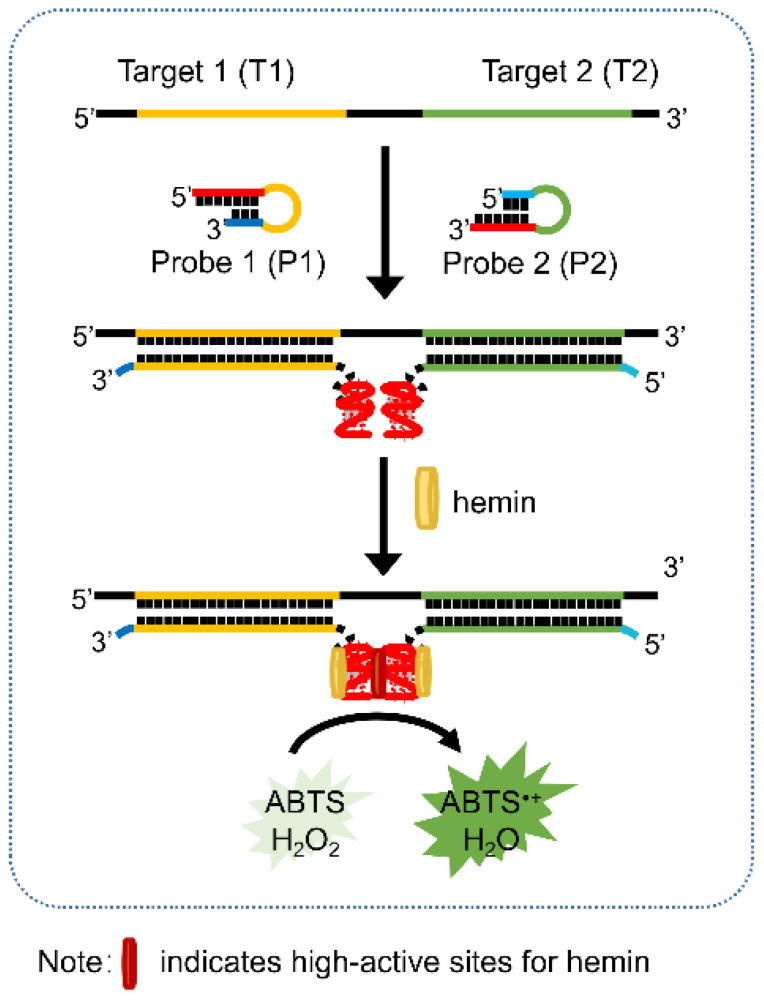
diG4/hemin DNAzyme biosensor. The hybridization of two probes with the adjacent targets allows the formation of a dimerized G-quadruplex and, thus, provides high-activity sites for hemin so as to enhance the catalytic activity of the DNAzyme.

In this study, based on the high catalytic efficiency of the multimeric G-quadruplex, we developed a high-sensitivity dimeric G-quadruplex/hemin DNAzyme (diG4/hemin DNAzyme) biosensor targeting Norovirus nucleic acids. As illustrated in Scheme 1, two molecular beacons (Probe 1 and Probe 2, abbreviated as P1 and P2) targeting adjacent DNA strands (Target 1 and Target 2, abbreviated as T1 and T2) were rationally designed with G-quadruplex-forming sequences, each embedded in the stem structure. As P1 and P2 hybridized with the adjacent T1 and T2, a three-way junction architecture formed, which could bring two released G-quadruplexes to dimerize, and thus, upon binding with hemin, could synergistically enhance the capacity to catalyze the H_2_O_2_-mediated oxidation of slightly green colored ABTS to visible green radical ABTS+. In our optimized reaction system, the colorimetric readout of target Norovirus conserved sequence was as low as 10 nM. Hence, our approach set up a non-amplifying, non-labeling, simple-operating, cost-effective colorimetric biosensing method for the detection of target nucleic acids, and also proposed further implications of multimerization of G-quadruplex in terms of the improvement of DNAzyme-based biosensor sensitivity.

## 2. Experimental Section

### 2.1. Materials and Reagents

The oligodeoxynucleotides (ODNs) corresponding to the norovirus conserved sequence target and the designed probes are listed in Table 1. All the ODNs were synthesized and ultrapage-purified by Sangon Biotech (Shanghai, China) without further desalting, dissolved in Milli-Q water and quantified using a Thermo Scientific Nanodrop 2000 spectrophotometer (Waltham, MA, US) to generate a stock solution at 100 μM. ABTS and hemin were purchased from Yuanye Bio-Tech (Shanghai, China). H_2_O_2_ (30%) was purchased from Damao Chemical Reagents (Tianjin, China). The stock solution of ABTS (40 mM) or H_2_O_2_ (40 mM) was prepared in Milli-Q water and that of hemin (100 μM) was prepared in DMSO and stored in dark at 4 °C. All the other reagents were of analytical grade and purchased from Sangon Biotech (Shanghai, China).

### 2.2. Colorimetric Detection of Target ODNs

The designed probes and corresponding target ODNs were mixed in a buffer containing 20 mM MgCl_2_, 100 mM Tris-HCl (pH 7.4) with 0–100 mM KCl or NH_4_Cl at described concentrations. The mixtures were heated at 95 °C for 5 min to dissociate any secondary structures and gradually cooled down to 25 °C for at least 2 h using a gradual cooling (0.1 °C/30 s) procedure. Afterwards, 2 μM hemin was added to the annealed mixture, which was subsequently incubated at 37 °C for 1 h to form the G-quadruplex/hemin DNAzyme. Finally, 4 mM ABTS and 4 mM H_2_O_2_ were added to the generated DNAzyme solution and mixed thoroughly. The color change produced by the ABTS-H_2_O_2_ reaction was observed and captured using a cell phone camera afterwards.

### 2.3. Circular Dichroism (CD) Spectroscopy

CD experiments were performed with a JASCO J-815 CD spectrometer (Tokyo, Japan) to evaluate the conformation of the designed probes in the presence or absence of corresponding targets. Measurements were conducted in a 0.1 cm path-length cuvette with a scanning wavelength range from 400 to 200 nm at 1 nm intervals. Three scans were averaged for each spectrum.

### 2.4. Gel Electrophoresis

Polyacrylamide gel electrophoresis (PAGE) experiments were performed for the separation of the probes with or without their corresponding targets. Nondenaturing PAGE was performed on a 15% polyacrylamide (Acrylamide/Bis 19:1) gel in 1×TBE buffer containing 90 mM Tris-Borate and 2 mM EDTA (pH = 8.0). ODN samples (8 μL) were loaded with 2 μL 5× sucrose gel loading buffer and 4S Redplus nucleic acid dye was used for staining in the dark for 40 min. The electrophoresis was run at 90V for 2 h and imaged using a BioRad Gel Doc XR+ system (Hercules, CA, USA).

### 2.5. Ultraviolet (UV) Spectroscopy

The designed probes, in the presence or absence of corresponding targets, were treated using the described colorimetric detection method and 8 min after the addition of ABTS and H_2_O_2_, the UV absorbance was collected using a JASCO V-750 UV-Visible Spectrophotometer (Tokyo, Japan). Samples were tested in a 0.1 cm path-length cuvette and data were collected from 500 to 400 nm at 1 nm intervals with a scanning speed of 100 nm/min. Each spectrum represents an average of two scans.

## 3. Results and Discussion

### 3.1. Design and Optimization of Colorimetric Buffer and Probes

**Figure 1:**
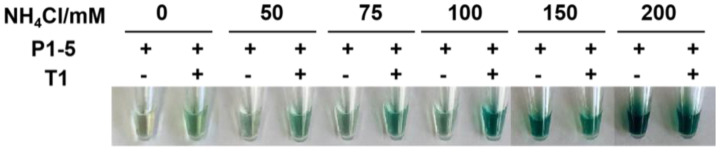
Photograph of colorimetric detection of target T1 by probe P1-5 at different concentrations of ammonium ions. Conditions: 2 μM of each of the probe or target, 20 mM MgCl_2_, 100 mM Tris-HCl (pH 7.4), and 0–200 mM NH_4_Cl, as illustrated.

Taking the conserved sequence linking orf1 and orf2 of norovirus GI [26] (Norwalk virus 8FIIa strain, GenBank ID: m87661: 5303-5362) as a target, we designed two molecular beacon probes—P1 and P2. In each of the probes, a widely used parallel G-quadruplex-forming sequence PW17 (GGGTAGGGCGGGTTGGG) [27] was chosen as the reporter sequence, and a short complementary sequence with 5, 6 or 7 nucleotides (see Table 1, P1-5/6/7 or P2-5/6/7) was designed to form a short stem with the G-rich sequence so as to block the G-quadruplex structure in the absence of the target. In order to promote the duplex formation either by the stem of the probe itself or the hybridization of the probe with the target, magnesium chloride was used in the colorimetric buffer [28]. As shown in Figure 1 (the very left panel), in the absence of the target, the probe P1-5 showed no catalytic activity for oxidation of ABTS, indicating that it folded into a hairpin structure which blocked the reporter sequence from forming G-quadruplex; however, in the presence of the target T1, although no additional monovalent cations were added to the colorimetric buffer, light yet visible green-colored radical ABTS+ was formed, which indicated the release of the G-rich sequence from the probe through hybridization with the target and partial formation of the G-quadruplex structure, since magnesium ions are reported to be able to bind and stabilize G-quadruplexes to some extent [29]. To further induce and stabilize the released G-quadruplex structure in the presence of the target, monovalent ions were introduced into the colorimetric buffer. At first, potassium chloride was used, but it can cause a high background in the probe itself (Figure S1), which may be due to the fact that the G-quadruplex structure in the presence of potassium ions is thermodynamically more favored than the designed hairpin structure. Thus, we tested ammonium chloride, which was previously reported to be able to enhance the colorimetric performance in the H_2_O_2_-ABTS reaction catalyzed by G-quadruplex/hemin DNAzyme [30]. Our results showed that the ammonium ions could significantly enhance the signal as the NH_4_^+^ concentration increased, and produced a slightly visible green background for the probe itself when the concentration was no more than 100 mM (Figure 1). Hence, the colorimetric buffer containing 100 mM NH_4_^+^ was used in the following probes and dimeric G-quadruplex/hemin DNAzyme system optimization.

**Figure 2:**
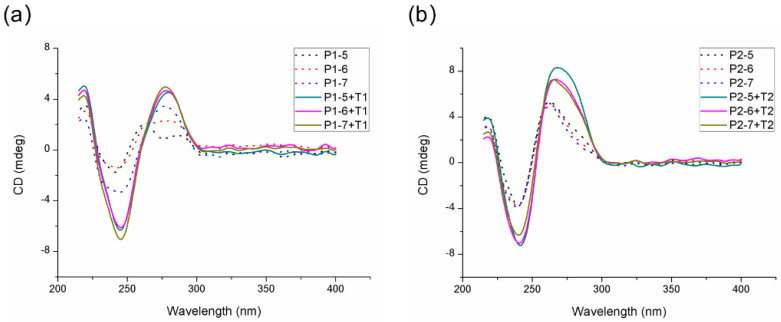
CD spectra of the designed probes (**a**) P1 and (**b**) P2 with different stem lengths (5, 6 and 7 bp) in the absence and presence of the corresponding targets T1 and T2, respectively. Conditions: 2 μM for each of the probes or targets, 20 mM MgCl_2_, 100 mM NH_4_Cl, and 100 mM Tris-HCl (pH 7.4).

To obtain an optimized signal-to-noise level, probes with varied stem lengths (5 bp, 6 bp and 7 bp) were evaluated. We first investigated the structure of the designed probes in the absence or presence of the corresponding targets using CD spectroscopy. As shown in Figure 2, the designed probes P1 (Figure 2a) and P2 (Figure 2b), with different stem lengths (5, 6 and 7 bp), had positive absorption around 260 nm and 280 nm in CD spectra (the dashed lines), indicating that all the probes could form DNA hairpin structures [31,32], which was consistent with the DNA secondary structure prediction result (Figure S2); while when the corresponding target T1 or T2 were added to the designed probes, the CD spectra showed much stronger and broader absorption around 260 nm to 280 nm, which was characteristic of duplex DNA and also overlapped with the absorption of parallel G-quadruplex DNA. We also evaluated the thermal stability of the probes with or without their corresponding targets. The results (Figure S3) show that the CD melting temperature (T_m_) of the probe in the presence of the target was 3 °C higher than that without the target, which was likely caused by the hybridization of the probe with its corresponding target and release of the G-quadruplex reporter sequence. Furthermore, we evaluated the structure of the designed probes in the absence or presence of the corresponding targets in the condition with hemin and the results show that the structures remained unchanged after the addition of hemin since the CD absorption remained more or less the same as before (Figure S4).

**Figure 3:**
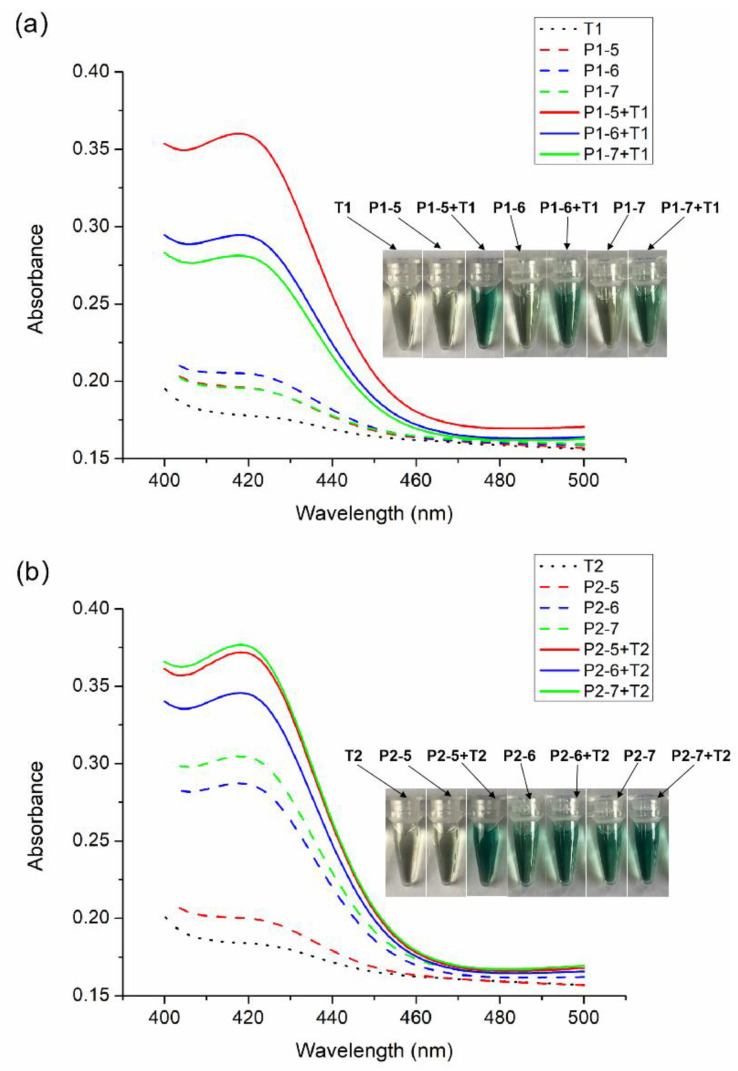
The UV spectra and colorimetric photograph (inner panel) of the individual probes (**a**) P1 and (**b**) P2 with different stem lengths (5, 6 and 7 bp) in the absence or presence of the corresponding targets. Conditions: 2 μM for each of the probes or targets, 20 mM MgCl_2_, 100 mM NH_4_Cl, 100 mM Tris-HCl (pH 7.4), 2 μM hemin, 4 mM H_2_O_2_, and 4 mM ABTS.

We next evaluated the performance of the individual probes through UV spectroscopy and naked-eye observation. As illustrated in Figure 3a, the target T1 itself barely caused UV absorbance in the H_2_O_2_-ABTS reaction (the dot line), and the probes P1-5, P1-6 and P1-7 all showed low background (the dashed lines) and were nearly colorless by naked-eye observation (the inner tubes P1-5, P1-6 and P1-7). In the presence of the target T1, the probe P1-5 showed high catalytic efficiency for the oxidation of ABTS by H_2_O_2_ and, thus, generated a strong UV absorbance at 417 nm (the red solid line) and an obvious visible green color (the inner tube P1-5 + T1), while the probes P1-6 and P1-7 exhibited relatively lower catalytic activities as the UV absorbance at 417 nm was much lower (the blue and green solid lines) and the visible green color was much weaker (the inner tube P1-6 + T1 and P1-7 + T1) compared with that of P1-5. This may have been due to the fact that after hybridization with T1, the probe P1, bearing a 6- or 7-nt stem, released a longer flexible 3′ toehold, which may have impaired the stability of the architecture and thus led to lowered signal. For probe 2, as shown in Figure 3b, the corresponding target T2 barely generated UV absorbance (the dot line) and was nearly colorless (the inner tube T2). Although all the probes, with different stem lengths (P2-5, P2-6 and P2-7), showed high detection signal in the presence of T2, which can be seen from the high UV absorbance (the red, blue and green solid lines) and strong visible green color (the inner tubes P2-5 + T2, P2-6 + T2 and P2-7 + T2), the backgrounds of P2-6 and P2-7 themselves were too high to be distinguished from the signal (the blue and green dashed lines, and the inner tubes P2-6 and P2-7), while that of P2-5 was much lower (the red dashed line and the inner tube P2-5). Although longer stems are supposed to block the probe more tightly [33], P2 with a 5-nt stem (P2-5) holds a 3′ toehold consisting of three entire GGG tracts, which was able to form G-triplex structure that may have hindered the opening of the stem and hampered it from refolding into a parallel G-quadruplex [34], thus generating a lower background than that obtained with a 6- or 7-nt stem. Above all, the individual probes P1 and P2 were both optimized to have a stem length of 5 bp (P1-5 and P2-5) in order to obtain relatively high signal-to-background levels in the biosensor design.

### 3.2. diG4/Hemin DNAzyme Optimization

**Figure 4:**
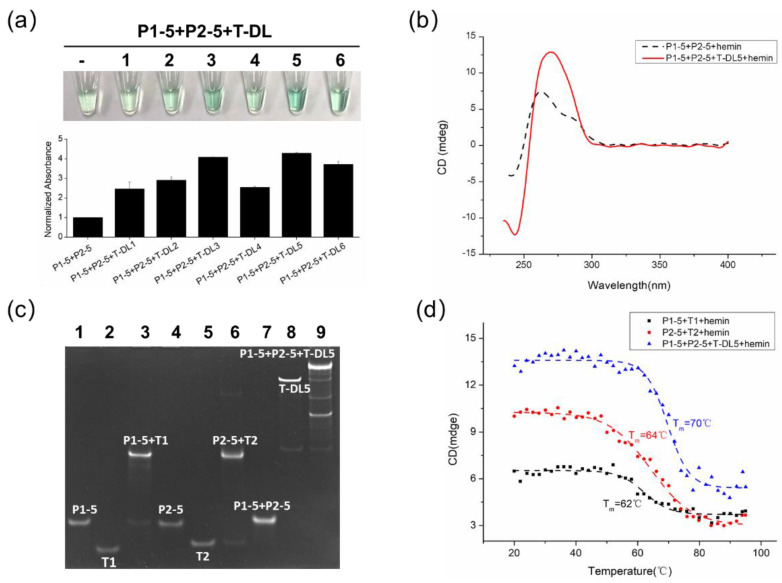
(**a**) Colorimetric photograph (upper) and normalized absorbance (bottom) of 1 μM of the molecular beacons P1-5 and P2-5 in the absence or presence of 0.05 μM of each of the targets with two adjacent hybridization sites separated by 6 to 1 nt. (**b**) CD spectra of 1 μM of the molecular beacons P1-5 and P2-5 in the absence or presence of 1 μM T-DL5 followed by the addition of 2 μM hemin in the colorimetric buffer. (**c**) The electrophoresis band of the molecular beacons (line 1: P1-5; line 2: P2-5), the separated targets (line 2: T1; line 5: T2), the optimized target consisting of two adjacent hybridization sites separated by 5 nt (line 8: T-DL5) and the mixture of the molecular beacons with their corresponding targets (line 3: P1-5 + T1; line 6: P2-5 + T2; line 9: P1-5 + P2-5 + T-DL5). All the samples were prepared in colorimetric buffer and the concentration of each of the ODN was 1 μM. (**d**) The CD melting curve of 1 μM of P1-5 and P2-5 with 1 μM of their corresponding separated targets or the optimized target T-DL5 followed by the addition of 2 μM hemin in the colorimetric buffer.

The distance between the two adjacent molecular beacons affected the stacked dimeric G-quadruplex structure in the presence of the target. As each of the reporter G-quadruplex has three G-tetrad layers, the maximum distance raised by the stacking of the two reporter G-quadruplex should have been no more than 6-nt, and thus, the initially selected target (T-DL6) consisted of two adjacent hybridization sites (T1 and T2) for each of the two probes that were 6-nt apart. We first evaluated the detection of the target T-DL6 by the dual probes P1-5 and P2-5 through UV absorbance. As shown in Figure S5, the UV absorbance of the target with the dual probes (red line) was around 30% higher than the sum of that of the individual probes (green line), which indicated that there was a synergetic effect between the two probes. In order to form a more efficient stacked dimeric G-quadruplex structure that had enhanced catalytic activity, the distance between the two probes was further optimized. We simply truncated the distance between the two selected targets to 5, 4, 3, 2 and 1-nt, and thus generated five truncated targets (T-DL5, T-DL4, T-DL3, T-DL2 and T-DL1) and evaluated their corresponding colorimetric detection using the previously optimized P1-5 and P2-5. As shown in Figure 4a, the target T-DL5, which corresponded to the distance of 5 nt between the two adjacent probes, exhibited the most intense visible green color, indicating that the G-rich sequences released from the hybridization of the two probes with the corresponding targets effectively formed stacked dimeric G-quadruplexes when the two probes were 5-nt apart. It is notable that when the distance was condensed to 4 nt, the catalytic activity of the diG4/hemin DNAzyme decreased significantly and, thus, a much weaker green color was detected for T-DL4 compared to T-DL5 and T-DL6. This may have been due to the fact that the two G-quadruplex motifs released from the adjacent probes were excluded from stacking with each other since the 5′ ends of P1 and 3′ end of P2 were too close. We further performed CD experiments and electrophoresis assays to evaluate the formation of the stacked dimeric G-quadruplexes through hybridization of P1-5 and P2-5 with T-DL5. As shown in Figure 4b, in the absence of the target, the mixture of the two probes (P1-5 + P2-5 + hemin) had two positive absorption bands near 260 nm and 280 nm, which was consistent with the CD absorption of each hairpin-structured probe. However, in the presence of the target T-DL5 (P1-5 + P2-5 + T-DL5 + hemin), the CD spectrum shows a much strong positive absorption at 267nm, indicating the formation of the long duplex structure by the hybridization of the probes P1-5 and P2-5 with the target T-DL5. This was supported by the electrophoresis results. As shown in Figure 4c, the individual probes P1-5 and P2-5 hybridized with their corresponding targets T1 and T2 (line 3 and line 6), and thus, migrated much more slowly than the separated probes (line 1 and line 4) or targets (line 2 and line 5). When the optimized probes P1-5 and P2-5 (line 7) were mixed with the target T-DL5 (line 8), a bright band corresponding to the long duplex formed by hybridization of the two probes with T-DL5 appeared (line 9). Furthermore, the thermal stability of the complexes formed by the hybridization of the individual probes P1-5 or P2-5 with their corresponding targets T1 or T2 were relatively close, with the CD melting temperature (T_m_) of 62 °C and 64 °C, respectively (Figure 4d, black line and red line), while the complex of P1-5 and P2-5 with T-DL5 was much more stable, with the T_m_ increasing significantly to 70 °C (Figure 4d, blue line), indicating the formation of the end-stacked dimeric G-quadruplexes that were released and brought together through the hybridization of the two adjacent probes P1-5 and P2-5 with the target T-DL5.

### 3.3. Detection Limitation of the diG4/Hemin DNAzyme Biosensor

**Figure 5:**
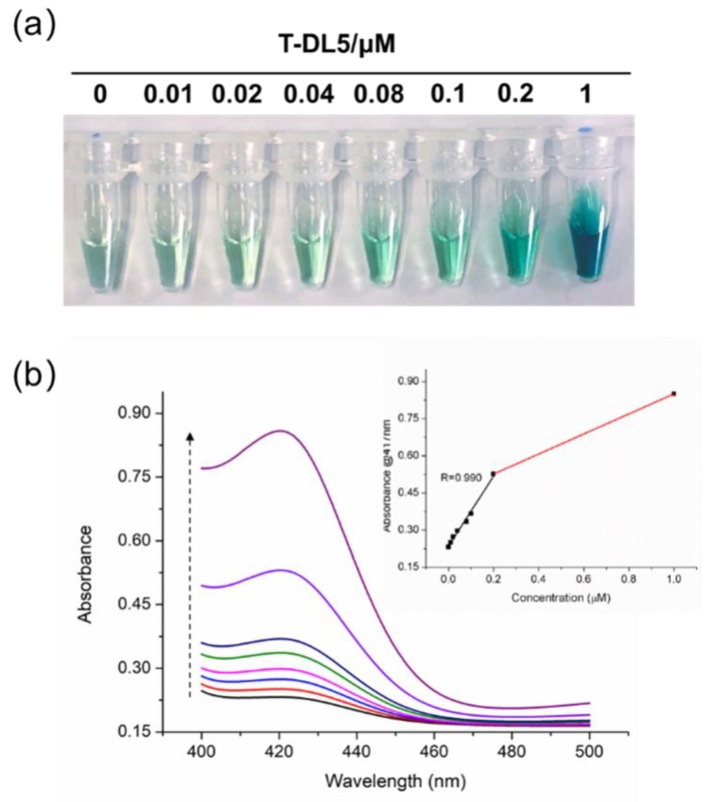
(**a**) Colorimetric observation and (**b**) UV absorbance of 0–1 μM T-DL5 in the presence of 1 μM P1-5 and 1 μM P2-5 in colorimetric buffer. The inner panel in (**b**) illustrates the linear fitting of UV absorbance at 417 nm with 0–0.2 μM T-DL5.

To further evaluate the sensitivity of the optimized diG4/hemin DNAzyme biosensor, we investigated the colorimetric limitation of the target T-DL5 by the probes P1-5 and P2-5 through naked-eye observation. As shown in Figure 5a, the visible green color faded as the concentration of the T-DL5 decreased, and the target was distinguishable from the background with a concentration as low as 0.02 μM. We further measured the UV absorbance of the samples with different concentrations of T-DL5; as shown in Figure 5b, the UV absorbance gradiently increased as the T-DL5 concentration increased, and the inner panel illustrates that the UV absorbance at 417nm was in linear correlation with the T-DL5 concentration when it was no more than 0.2 μM.

**Figure 6:**
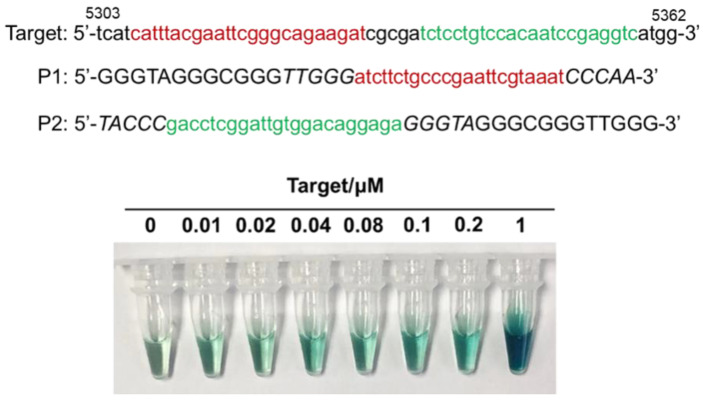
Sequences of the targeted norovirus GI conserved sequence (Target) and the two molecular beacons (P1 and P2), and the colorimetric observation of the target at different concentrations by 1 μM P1 and 1 μM P2 in colorimetric buffer.

Based on the probe and adjacent target distance optimization result, we finally designed two molecular beacons, P1 and P2, which targeted two adjacent sequences that were 5-nt apart within the norovirus GI-conserved sequence (GenBank ID: m87661: 5303–5362). As shown in Figure 6, we evaluated the colorimetric detection limitation of the designed biosensor and the results showed that the target sequence could be distinguished by naked-eye observation at 0.01 μM.

## 4. Conclusions

In this study, we developed a diG4/hemin DNAzyme biosensor utilizing norovirus as a model target. Nowadays, as the threat of various organisms and viruses against humans is becoming a prominent problem for public health [35], developing new DNA-based detection systems that utilize oligonucleotide itself as a reporter to generate visible signals is of great importance for the diagnosis of multiple virus infections. Here, we reported an easy methodology based on the high catalytic efficiency of stacked dimeric G-quadruplex/hemin DNAzyme through the rational design of two adjacent molecular beacon probes and applied it in the colorimetric detection of norovirus GI. Our biosensor has the advantages of non-labeling, non-protein enzyme involving, low cost and easy construction of probes for the detection assay, and it can currently detect target ODNs up to 10 nM through naked-eye observation. By easily substituting the hybridization segment of the probe to the sequence complementary of the target, this platform can be adapted for various nucleic acid detection assays of other viruses. Although our approach is more sensitive than the previously reported PCR-free, simple operational split G-quadruplex/hemin DNAzyme detection assays for dengue virus [36], its ability to detect actual virus mRNA might still be limited and further validation of the assay in various norovirus-infected samples is also required. In further studies, the diG4/hemin DNAzyme might be combined with non-enzymatic DNA amplification techniques such as catalyzed hairpin assembly (CHA) so as to further improve the sensitivity of measurement, and thus, to provide a simple, cost-effective and high-sensitivity biosensing system to promote the early screening of communicable diseases such as acute gastroenteritis caused by norovirus in underdeveloped areas.

## Figures and Tables

**Table 1:** The sequences of the ODNs used in this study.

Name	Sequences (5′-3′)
Target (T-DL6)	tcatcatttacgaattcgggcagaagatcgcgatctcctgtccacaatccgaggtcatgg
T1	tcatcatttacgaattcgggcagaagatcgcga
T2	tcgcgatctcctgtccacaatccgaggtcatgg
P1-5	GGGTAGGGCGGG*TTGGG*tcttctgcccgaattcgtaaatg*CCCAA*
P2-5	*TACCC*gacctcggattgtggacaggaga*GGGTA*GGGCGGGTTGGG
P1-6	GGGTAGGGCGG*GTTGGG*tcttctgcccgaattcgtaaatg*CCCAAC*
P2-6	*CTACCC*gacctcggattgtggacaggaga*GGGTAG*GGCGGGTTGGG
P1-7	GGGTAGGGCG*GGTTGGG*tcttctgcccgaattcgtaaatg*CCCAACC*
P2-7	*CCTACCC*gacctcggattgtggacaggaga*GGGTAGG*GCGGGTTGGG
T-DL5	tcatcatttacgaattcgggcagaagatcgcgtctcctgtccacaatccgaggtcatgg
T-DL4	tcatcatttacgaattcgggcagaagatcgctctcctgtccacaatccgaggtcatgg
T-DL3	tcatcatttacgaattcgggcagaagatcgtctcctgtccacaatccgaggtcatgg
T-DL2	tcatcatttacgaattcgggcagaagatctctcctgtccacaatccgaggtcatgg
T-DL1	tcatcatttacgaattcgggcagaagattctcctgtccacaatccgaggtcatgg

## Data Availability

Not applicable.

## Supplementary Materials

The following are available online. Additional figures are noted in the text. Figure S1: Photograph of colorimetric detection of target T1 by probe P1-5 at different concentrations of potassium ions. Conditions: two micrometers of each of the probes or targets, 20 mM MgCl_2_, 100 mM Tris-HCl (pH 7.4), 0–50 mM KCl, as illustrated; Figure S2: The predicted secondary structure of the designed probes using the online software RNAstructure (http://rna.urmc.rochester.edu/RNAstructureWeb/ accessed on 18 January 2021). Figure S3: (a) the CD melting curve and (b) melting temperature of the probe P1-5 or P2-5 in the presence and absence of their corresponding targets T1 or T2. Conditions: two micrometers for each of the probes or targets, 20 mM MgCl2, 100 mM NH4Cl, 100 mM Tris-HCl (pH 7.4); Figure S4: CD spectra of the designed probes (a) P1 and (b) P2 in the absence and presence of their corresponding targets T1 and T2 in the condition with hemin. Conditions: two micrometers for each of the probes or targets, 20 mM MgCl2, 100 mM NH4Cl, 100 mM Tris-HCl (pH 7.4), 2 μM hemin. It is notable that the hemin was dissolved in DMSO, which usually causes a high background around 200 to 230 nm in the CD spectrum, so the scanning wavelength ranged from 235 to 400 nm. For a clear comparison, the corresponding CD spectra without hemin are also presented here as (c) and (d); Figure S5: (a) UV absorbance of the dual probes or the individual probes in the absence or presence of the target T-DL6. The green line shows the calculated sum UV absorbance of the individual probes with the target. (b) Comparison of the UV absorbance at 417 nm of the dual probes or the individual probes in the presence of the target.
