# Supplementary material for: Design of a High-Sensitivity Dimeric G-Quadruplex/Hemin DNAzyme Biosensor for Norovirus Detection
Source: Molecules. 2021 Dec 3;26(23):7352. doi: 10.3390/molecules26237352 (PMC8659037; doi:10.3390/molecules26237352)
Supplement: Supplementary file 1 [file molecules-26-07352-s001.zip › molecules-1470296-supplementary.pdf]

Design of a High-Sensitivity Dimeric G-Quadruplex/ Hemin DNAzyme Biosensor for Norovirus Detection

Yun Zhang [1,†], Xinao Ma [1,†], Jingtian Zhang [1], Feixian Luo [1,2], Wenshu Wang [1,2] and Xiaojie Cui [1,2,*]

[1] College of Life and Environmental Sciences, Minzu University of China, Beijing 100081, China
[2] Key Laboratory of Ecology and Environment in Minority Areas (Minzu University of China), National Ethnic Affairs Commission, Beijing 100081, China
* Correspondence: xiaojiecui@muc.edu.cn
† These authors contributed equally to this work.

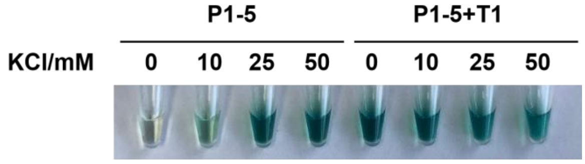

Figure S1. Photograph of colorimetric detection of target T1 by probe P1-5 in different concentration of potassium ions. Conditions: 2 uM of each of the probe or target, 20 mM $MgCl_2$, 100 mM Tris-HCl (pH 7.4), 0-50 mM KCl as illustrated.

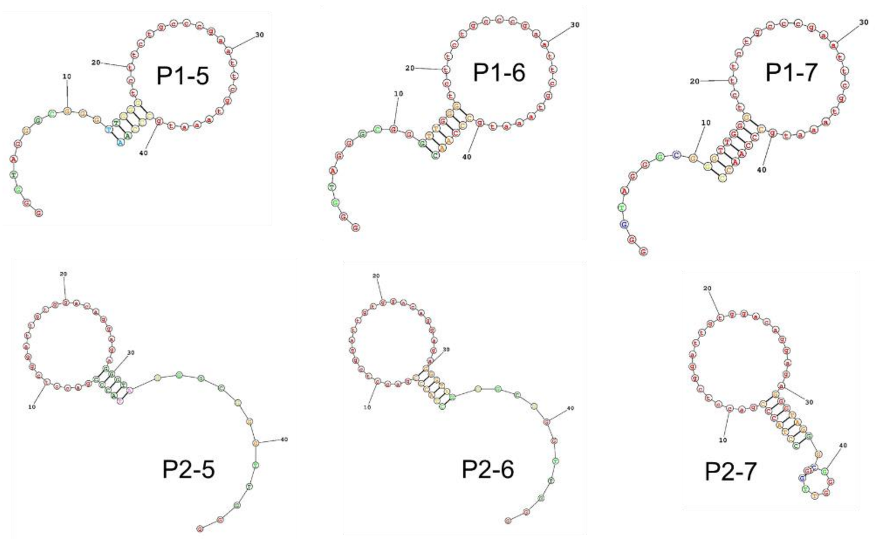

Figure S2. The predicted secondary structure of the designed probes by online software RNAstructure (http://rna.urmc.rochester.edu/RNAstructureWeb/).

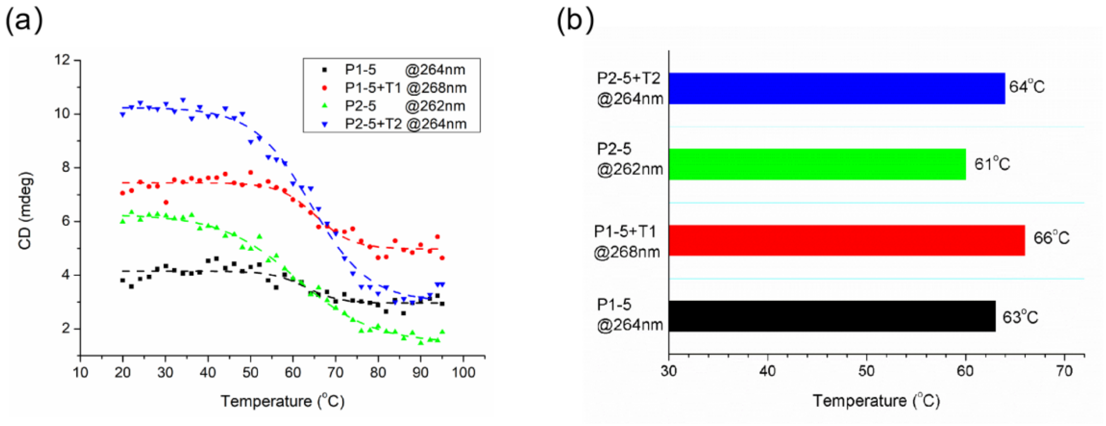

Figure S3. (**a**) the CD melting curve and (**b**) melting temperature of the probe P1-5 or P2-5 in the presence and absence of their corresponding target T1 or T2. Conditions: 2 uM for each of the probes or targets, 20 mM $MgCl_2$, 100 mM NH_4Cl, 100 mM Tris-HCl (pH 7.4).

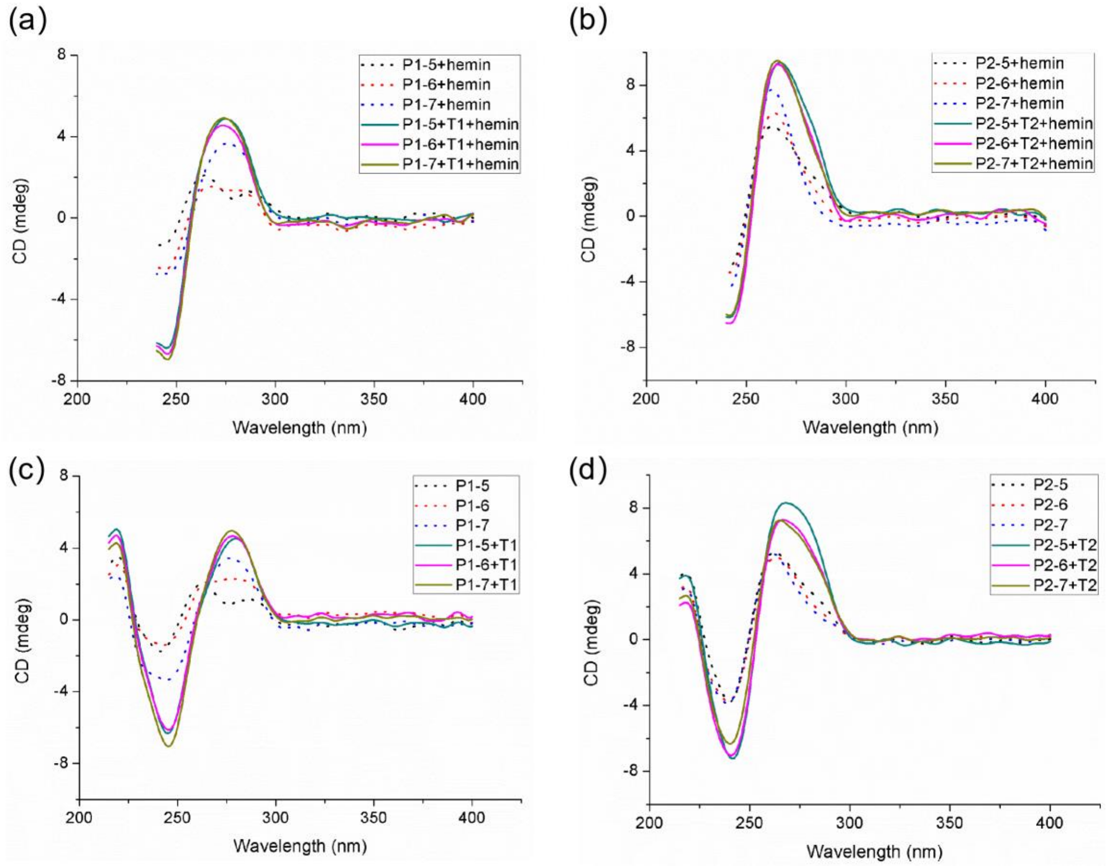

Figure S4. CD spectra of the designed probes (**a**) P1 and (**b**) P2 in the absence and presence of the corresponding targets T1 and T2 in the condition with hemin. Conditions: 2 uM for each of the probes or targets, 20 mM MgCl2, 100 mM NH4Cl, 100 mM Tris-HCl (pH 7.4), 2 uM hemin. It is notable that hemin is dissolved in DMSO which usually causes a high background around 200 to 230 nm in the CD spectrum, so the scanning wavelength ranges from 235 to 400 nm. For a clear comparison, the corresponding CD spectra without hemin are also presented here as (**c**) and (**d**).

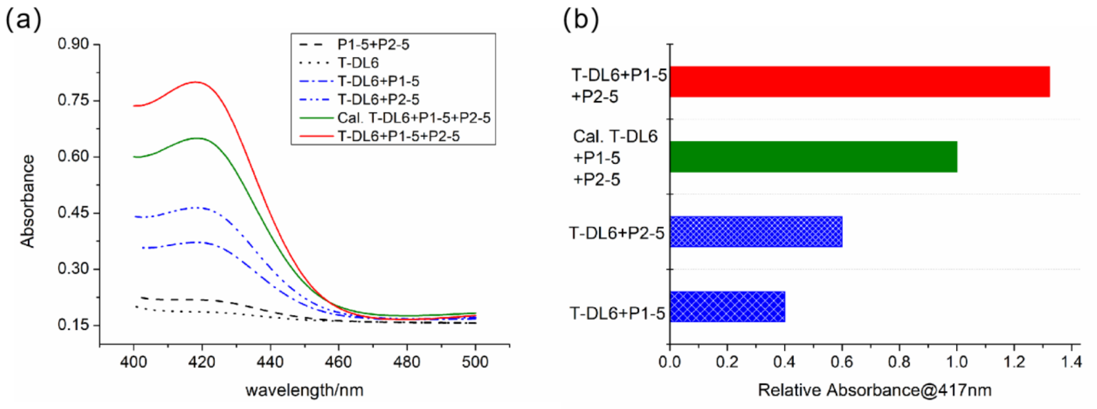

Figure S5. (**a**) UV absorbance of the dual probes or the individual probes in the absence or presence of the target T-DL6. The green line shows the calculated sum UV absorbance of the individual probes with the target. (**b**) Comparison of the UV absorbance at 417 nm of the dual probes or the individual probes in the presence of the target.
